# Key Characteristics and Mechanisms in Ventilator Weaning of Adult Intensive Care Patients—A Qualitative Study With Healthcare Professionals

**DOI:** 10.1111/nicc.70678

**Published:** 2026-09-24

**Authors:** Fritz Sterr, Lydia Bauernfeind, Anja Kepplinger, Christian Rester, Rebecca Palm, Sabine Metzing

**Affiliations:** ^1^ Faculty of Health, School of Nursing Sciences Witten/Herdecke University Witten Germany; ^2^ Faculty of Applied Healthcare Sciences Deggendorf Institute of Technology Deggendorf Germany; ^3^ Department of Health Services Research, School VI Medicine and Health Sciences Carl von Ossietzky Universität Oldenburg Oldenburg Germany

**Keywords:** artificial respiration, critical care, intensive care, mechanical ventilation, program theory, qualitative study, ventilator weaning

## Abstract

**Background:**

Weaning from mechanical ventilation appears to be a complex intervention as defined by the Medical Research Council Framework. However, there is a lack of theoretical insights into ventilator weaning, and the underlying causal mechanisms have not yet been uncovered sufficiently. To understand its complexity in theory and develop it in practice, a program theory is currently being developed in a multi‐method study.

**Aim:**

To identify key characteristics and mechanisms in ventilator weaning of adult intensive care patients.

**Study Design:**

In this sub‐study, we conducted semi‐structured group discussions and workshops with 29 healthcare professionals in intensive care over 3 days in 2025 in Germany, using a qualitative design. The data were collected using audio recordings, field notes and photographs. After transcription, a deductive‐inductive thematic analysis was performed.

**Findings:**

The analysis revealed four principal themes: (1) complexity of ventilator weaning, (2) outcomes, (3) interventions and (4) links and relationships. The first theme combines factors such as definition and delimitation, dynamics, structure and individualization, which together describe the inherent complexity. The second theme covers different endpoints during the process, including preconditions and postconditions as well as intermediate, immediate and ultimate outcomes. The third theme comprises direct and indirect interventions, as well as nonprogram external factors. The fourth theme explores the connections between interventions, outcomes and contextual factors.

**Conclusion:**

The findings of this study support the understanding that ventilator weaning is a complex intervention. The identified interaction of the various interventions, outcomes and contextual factors needs to be theoretically organized and evaluated in future studies.

**Relevance to Clinical Practice:**

Identifying the interactions between key characteristics in this process can improve HCPs' sensitivity to the effects of their own actions. A theoretical understanding of the mechanisms underlying ventilator weaning enables the targeted development and evaluation of a complex weaning intervention.

**Trial Registration:**

Open Science Framework YGJ3T; https://doi.org/10.17605/OSF.IO/YGJ3T, registered on 14 May 2025

## Impact Statements

- What is known about the topic
  ○ Numerous empirical findings are available internationally on mechanical ventilation and its weaning.
  ○ Several interventions and outcomes are relevant in ventilator weaning.
- What this paper adds
  ○ Ventilator weaning is confirmed as a complex intervention based on various characteristics.
  ○ The study provides detailed information on relevant interventions, outcomes and contextual factors for developing a program theory.

**Abbreviations**
APN: advanced practice nurse
FN: fieldnote
GD: group discussion
HCP: healthcare professional
ICU: intensive care unit
L: line
LL: lines
MRC: medical research council
MV: mechanical ventilation
OSF: open science framework
PT: program theory
RN: registered nurse
RT: respiratory therapist
SBT: spontaneous breathing trial
SRQR: standards for reporting qualitative research
VW: ventilator weaning
WIND: weaning according to a new definition

## 1 Introduction

### 1.1 Background

Mechanical ventilation (MV) is used in intensive care units (ICUs) worldwide to stabilize patients with severe (respiratory) conditions. However, MV also carries considerable risks resulting in adverse events [1] and increased patient mortality [2] when used for prolonged periods. Early on during the MV, the question of ventilator weaning (VW) becomes relevant to protect patients from these risks.

Today, there is a large body of evidence on VW, which is also apparent in various guidelines for this process [3, 4]. These findings suggest that VW is not a simple but rather a complex intervention as defined by the Medical Research Council (MRC) framework [5]: It involves various healthcare professionals (HCPs) with different skills and degrees. VW targets different populations, depends on the underlying conditions of the patients and requires flexibility in action.

### 1.2 Problem Definition

Despite in‐depth knowledge in respiratory medicine, actual VW in ICUs is still inadequate [6]: Weaning failure rates are high, with 24.3% never entering the weaning process [2] and 28.3% dying while receiving MV [7]. In addition, more patients than assumed can be weaned but are declared ‘unweanable’ prematurely [8]. Central to this is a broad theoretical deficit in VW that is characterized by incomplete, fragmented interventional research. There is a lack of in‐depth understanding of the relationship between interventions and outcomes and how these are influenced by their respective contexts [9].

To address this research gap, a program theory (PT) for VW of adult ICU patients is currently being developed. The study follows the understanding and methodological recommendations of Funnell & Rogers [10], who describe a PT as ‘an explicit theory or model of how an intervention […] contributes to a chain of intermediate results and finally to the intended outcomes’ [10]. In line with the MRC Framework [5], the overall aim of this study was to explore the interconnectedness of interventions, outcomes and context in VW [9]. The study is divided into two steps: In the first step, an initial PT was developed based on literature, abduction and stakeholders. In the second step, the PT is reviewed and refined with HCPs [9]. This article presents the data obtained in a sub‐study for the revision of the second step.

## 2 Aim and Research Questions

The aim of this sub‐study was to identify key characteristics and mechanisms in VW of adult ICU patients. In this approach, characteristics comprise the core components of VW (interventions, outcomes and context) as well as their attributes. Mechanisms describe the interaction among these components and are understood as causal relationships between individual program activities, intermediate results and the final outcomes to be achieved. These, in turn, are embedded in their context and unfold their impact within it [10]. These findings are needed as a basis for revising the PT in the final step of the study.

It addresses the following research questions:

- What interventions are relevant to VW of adult intensive care patients?
- What outcomes are relevant to VW of adult intensive care patients?
- What contextual factors are relevant to VW of adult intensive care patients?
- How are interventions, outcomes and contextual factors interconnected in VW of adult intensive care patients?

## 3 Design and Methods

This qualitative study is embedded in the second step of a larger multi‐method study. It was registered in the Open Science Framework (OSF; ID YGJ3T) in May 2025, and a study protocol was published, which provides a comprehensive overview of the underlying problems and the methodological approach of the study [9].

This article reports the results of the data collection and analysis in Step 2 and serves as the basis for revising the PT. The revised version will then be presented in a subsequent article.

### 3.1 Qualitative Approach and Research Paradigm

Embedded in the logic of program theories, this study adopts an ontologically realist position combined with a pragmatic and context‐sensitive epistemological and methodological stance. Ontological realism assumes that structures, conditions and processes exist and operate independently of what we know and experience [11]. These phenomena are understood as situated and fallible at the epistemological level and may vary across cultures, conceptions or situational circumstances [11]. This positioning is compatible with the approach to program theory proposed by Funnell and Rogers [10], in which program theories provide explicit and revisable representations of how change is brought about in particular contexts. Accordingly, we combine realist assumptions about the existence of phenomena with a pragmatic approach to their conceptualization and investigation in line with the underlying methodology.

### 3.2 Researcher Characteristics and Reflexivity

Five authors of the study have previously worked in ICUs with MV patients (FS, LB, AK, CR and SaM), and one (LB) still works there part‐time. Both researchers (FS and LB) who primarily collected and analysed the data for this study were involved in developing the initial PT. In addition, both are familiar with a third of the participants through various forms of professional collaboration.

To counteract potential author bias, several strategies were employed. Prior to the first data collection, a comprehensive discussion was held with the entire team, during which the researchers articulated their initial assumptions and preconceptions. These were recorded in a research diary kept in parallel. Before and after each data collection session, briefing and debriefing sessions were also held between the lead researchers (FS and LB) and a third researcher (AK), who served as an independent auditor and was not involved in data collection or analysis. The procedures and perspectives of the two researchers were repeatedly disclosed to her and discussed with regard to potential influences. Finally, the study's findings were compared with the preconceptions articulated by the group at the outset to rule out any potential biases.

### 3.3 Eligibility Criteria and Sampling Strategy

We used purposive sampling to recruit participants for this study. Various HCPs were informed via professional networks of the study authors. To represent realistic staffing levels in ICUs, we sought to obtain a heterogeneous sample regarding professional experience, degrees, tasks and roles. To this end, we deliberately kept the inclusion criteria broad.

Specifically, HCPs were eligible to participate in this study if they had completed training in medicine, nursing, physical therapy, speech therapy or respiratory therapy and had at least 1 year of professional experience with MV patients in the ICU. In addition, they had to be 18 years of age or older and speak and understand German.

### 3.4 Characteristics of Study Participants

In total, 29 HCPs participated in the study. Of these, 89.6% were registered nurses (RN) and three were respiratory therapists (RT). Eight worked as ICU managers, three as teachers at a nursing school, 11 as ICU nurses, three as RTs in the ICU, three as mentors for trainees and one as an advanced practice nurse (APN). The participants had varying degrees of experience in intensive care, ranging from 1 year to 35 years. Sixty‐nine per cent were specialized in critical care, and 51.7% were trained mentors. Further characteristics of the study participants can be found in Table 1.

**TABLE 1:** Characteristics of study participants.

Study participants (*N* = 29)	
Age in years, mean (min‐max)	43.34 (21–60)
Vocational training	
Nursing, *n* (%)	26 (89.6%)
Respiratory therapy, *n* (%)	3 (10.3%)
Special training, *n* (%)	25 (86.2%)
Critical care, *n* (%)	20 (69%)
Mentor, *n* (%)	15 (51.7%)
Ward leader, *n* (%)	4 (13.8%)
Highest academic degree	
None, *n* (%)	19 (65.5%)
Bachelor, *n* (%)	7 (24.1%)
Master, *n* (%)	3 (10.3%)
Overall professional experience in years, mean (min‐max)	21.03 (2–40)
Years of experience in intensive care, mean (min‐max)	17.45 (1–35)

*Note:* This table contains the key characteristics of the 29 healthcare professionals who participated in the study.

### 3.5 Ethical Considerations

This study was conducted in accordance with the Declaration of Helsinki [12]. A data protection management procedure was initiated and approved at the study site on 24 March 2025. In addition, a positive ethics vote was issued by the Ethics Committee of Witten/Herdecke University on 28 April 2025 (Nr. S‐109/2025).

These applications stated that the participants did not belong to a vulnerable group. Nevertheless, it was agreed that all interested professionals have to sign an informed consent form confirming their voluntary participation. They were informed that they could withdraw from the study at any time until the data were published, without giving reasons and without suffering any disadvantage. All data collected were immediately pseudonymized and anonymized for third parties. After completion of the study, but no later than the end of 2026, all collected data and personal information will be irrevocably deleted. No compensation was provided for travel expenses or work absences. However, participants were provided with catering during the study.

### 3.6 Data Collection

As recommended by Funnell & Rogers [10], group discussions (GD) [13] and workshops [10] were held to identify key characteristics and mechanisms in VW. A total of three data collection sessions were held in summer 2025 in Germany, each lasting 4 h. Between seven and 11 individuals participated each day.

During the 90‐min GD, questions were asked about the preconditions for VW, relevant outcomes, key interventions and the connection between these aspects, based on a semi‐structured interview guide [9].

Afterwards, they were confronted with the initial PT into two workshops. In 45 min each, the participants were asked to evaluate and update the arrangement and selection of outcomes and interventions. In the workshops, the main categories of interventions (direct, indirect and nonprogram) and outcomes (preconditions, immediate, intermediate, ultimate and post‐condition) were displayed on A3 sheets. All terms contained in the initial PT [9] were printed on A4 sheets and handed out to the participants. After their allocations, the results were discussed, reviewed and adjusted with the group. Examples of workshop results can be found in File S1.

Once the participants had made adjustments, the theory was adapted accordingly and presented in modified form to the next study group. This ensured an iterative approach following the methodological recommendations [10]. Congruence was the criterion established for concluding data collection in this study and was understood as the consistency of the analyses and participants' statements with the findings of the program theory [10]. This is reflected in a consistent description of the central mechanisms in the VW process. As congruence was achieved after the third study group, no further data collection was necessary.

The GD was audio‐recorded, and photographs of the final workshop arrangements were taken. In addition, fieldnotes (FN) were taken by both researchers (FS and LB) for all data collection stages.

### 3.7 Data Analysis

After data collection, the audio files were transcribed manually in Microsoft Word (FS) and verified by a second researcher (LB). Finally, a comprehensive data set was available for analysis, consisting of three transcripts of the GD, 18 FN from the GD and workshops and 24 photographs of the workshop results.

These data were analysed using a deductive‐inductive thematic analysis according to Braun and Clark [14, 15]. The principal deductive themes were interventions (with sub‐themes direct, indirect and nonprogram), outcomes (with sub‐themes preconditions, immediate, intermediate, ultimate and postconditions) and links and relationships. All other principal and sub‐themes emerged during the analysis. To ensure an iterative process of theory development, data analysis was performed immediately after each data collection. The analysis was performed in MAXQDA (version 24) by two researchers (FS and LB). In the event of discrepancies, a third researcher (CR) was consulted.

### 3.8 Enhancing Trustworthiness

To ensure the quality of this study, we are guided by four quality criteria: credibility, transferability, dependability and confirmability [16]. *Credibility* was ensured through prolonged engagement (an iterative process spanning several months), reflexivity (explicating one's own assumptions and comparing the study results with them) and triangulation of methods (workshops and GDs) and data (field notes, audio recordings and photographs). *Transferability* was achieved through a thick description of the findings, a transparent presentation of the recruitment strategy, and a heterogeneous sample that reflected the real‐world setting of the ICU. *Dependability* was ensured through a detailed methodological description, transparency regarding registration (OSF; ID YGJ3T), a study protocol [9] and accompanying supplements, as well as reporting in accordance with the ‘Standards for Reporting Qualitative Research’ (SRQR) guideline [17]. Finally, *confirmability* was achieved through peer debriefing (role assignment within the team: each step carried out by at least two researchers [FS, LB], with discussions before and after each step with a neutral third party [AK, CR], and two supervisors providing ongoing guidance [RP, SaM]), member checking (an iterative process involving multiple confirmations of content and by participants) and reflexive journaling (ongoing protocol and research diary).

## 4 Findings

The analysis of the data identified a total of four principal themes: (1) complexity of ventilator weaning, (2) outcomes, (3) interventions and (4) links and relationships. These are presented with their respective sub‐themes in Table 2 and are described in detail below. Exemplary excerpts from the data analysis with additional codes and text passages are illustrated in File S2.

**TABLE 2:** Principal themes and sub‐themes derived in the data analysis.

Principal themes	Sub‐themes
Complexity of ventilator weaning	Common understanding within the care team
	Delimitation of ventilator weaning
	Patients' individual needs and demands
	Dynamics in the weaning process
	Weighing up in difficult situations
	Significance of the factor time
	Structure and individualization
Outcomes	Characteristics of outcomes
	Preconditions
	Immediate outcomes
	Intermediate outcomes
	Ultimate outcomes
	Postconditions
Interventions	Characteristics of interventions
	Preparatory interventions
	Direct interventions
	Indirect interventions
	Nonprogram factors
	Post‐program factors
Links and relationships	Organization and process
	Disease and condition
	ICU surroundings
	Breathing and ventilation
	Sedation, sleep and cognition
	Nutritional intake
	Stress development
	Mobility and muscles

*Note:* This table summarizes the principal themes and sub‐themes of the data analysis in this study.

### 4.1 Complexity of Ventilator Weaning

Data analysis suggested that VW is linked to complex circumstances. Participants described the importance of a common understanding within the care team, as weaning is an interprofessional responsibility. They pointed to the need for linguistic precision and common terminology.

Now the question would be how weaning is defined […] the topic of indications if I say I now have ALS and will then always be dependent on it then […]. GD3, RN18, L170‐173

The statement suggests that an understanding can also change depending on the situation and must be renegotiated in terms of patient's condition. This is also related to the delimitation of ventilator weaning. It is not just about breathing properly, but not every procedure is automatically part of the weaning process. The question arises as to where weaning begins and where it ends when embedding the process into the overall care provided in the ICU.

RN26 adds that MV cannot be seen in isolation, but that numerous aspects […] must also be considered. FN17, L83‐85

Participants also highlighted a persistent tension between structure and individualization. On the one hand, there is a clear plea not to just try things out. A structured approach is essential; all ventilated patients have overlaps and weaning can be divided into different stages. On the other hand, it is important to tailor weaning to the patient's needs. Tube and tracheostomy weaning differ and some patients even fall completely out of line. It is therefore difficult to standardize the entire weaning process.

Furthermore, patients develop individual needs and demands. As mentioned by the participants, patients are sensitive to their own sensations and want to be seen as the person behind MV. They recommend patient‐friendly weaning while being aware of controlling patients externally and therefore having a special responsibility and advocacy role. HCPs should be close to the patients but not overburden them. According to the participants, patients play a key role in determining the indication for weaning and need guidance throughout the weaning process.

The dynamics in the weaning process characterize the overall complexity. Patients' situation varies and changes over time; HCPs must react flexibly to their dynamic needs. Prolonged weaning has its ups and downs, and the course of the entire weaning process is not always fully predictable. In addition, knowledge, equipment or financing requires healthcare providers to adapt their care structure over the years.

In addition, the participants emphasized the significance of the factor ‘time’. HCPs stated that the initiation of weaning is often the biggest challenge, although it should be started at an early stage. Indication and initiation do not always take place simultaneously, and prolonged weaning is an ongoing complex situation.

Finally, HCPs are weighing up in difficult situations. While MV is unphysiological and unpleasant for the patient, HCPs may have to maintain MV and need to prioritize issues. Ongoing critical illness and weaning initiation do not contradict each other, but not all progress is automatically good for the patient. As interventions can also worsen the patients' situation, a well‐dosed combination of interventions is recommended.

Taking together, VW emerges as a dynamic, nonlinear process that requires continuous balancing between standardized procedures and individual patient needs.

### 4.2 Outcomes

Pointing to the characteristics of weaning‐associated outcomes, participants emphasized that weaning needs to be realistic and meaningful for both patients and HCPs. According to them, it is essential to identify and formulate relevant outcomes in the weaning process and to evaluate them regularly. Goals may change during treatment, and different outcomes may become relevant at different stages. At the same time, certain outcomes may be relevant at several stages, and it may be important to maintain them throughout the process.

However, achieving a goal does not mean that this aspect is no longer considered, but rather that it is essentially complete but still important (e.g., if I have achieved adequate fluid balance as an immediate goal, this does not mean that I do not still have to consider it in the long term). FN12, LL74‐78

Participants mentioned preconditions that need to be addressed before VW can be initiated. They discussed an indication for weaning. This can differ among patients and coexist with the indication for MV. Furthermore, the indication for weaning must be distinguished from the indication for extubation. Finally, contraindications for weaning must also be considered.

I think there are many contraindications, i.e., if the patient has no spontaneous respiratory drive at all or […] if he has a fever of 40°C for days […] then of course it doesn't make sense because I use up a lot more of the patient's resources than the weaning attempt might actually benefit me. (GD2, RN08, LL257‐262)

In addition, weaning readiness is a key precondition. This is characterized by stable haemodynamics and vital signs, spontaneous activity, wakefulness and calmness as well as the presence of protective reflexes. However, the participants also note that vigilance per se is not a precondition and that weaning can be initiated despite supposed limitations.

I mean […] he just doesn't have to be vigilant but has his own respiratory drive (several participants agree). (GD3, RN21, LL609‐610)

The participants also named the readiness of the respiratory system as a precondition. In their opinion, this includes low tracheal secretion, the ability to breathe spontaneously, sufficient gas exchange and the level of respiratory pressure.

First I look at what respiratory pressures he has at all, what is my PEEP, can I even go into weaning, what are my values for this, so we reduce first and from a certain pressure or PEEP we enter weaning. (GD1, RN02, LL35‐37)

Furthermore, the participants mentioned negative predictors of weaning failure. Although this was classified as an immediate outcome by the first group, the subsequent groups differentiated between predictors for weaning and predictors for extubation. The group consensus was that the predictors for weaning are a precondition for initiating weaning.

The spontaneous breathing trial […] helps me to discriminate can I extubate him so can I wean him or can I not. (GD1, RT01, LL232‐234)

### 4.3 Interventions

The participants identified a variety of interventions and influencing factors in VW. These were categorized into direct and indirect interventions as well as nonprogram external factors. In addition, the participants described characteristics of the interventions and further distinguished between preparatory interventions before initiating the program and post‐program interventions after completion of VW.

Considering the characteristics, participants noted differences of interventions in terms of their timing, personnel resources and extent. They emphasized that gradual weaning is recommended and that even small steps are important for the process.

If, after three hours, I can reduce a parameter a little bit or perhaps reduce the FiO2 by five percent, then I've already achieved something in terms of weaning, even if it's only very small steps. (GD2, RN08, LL437‐439)

Although there is a major workload in the beginning, HCPs advised cautious initiation. The interventions to be performed depend on the underlying disease and should be combined in intervention bundles.

It is not the individual measure but the combination of all of them in bundles that is significant and relevant; there is not *the one* weaning intervention. (FN03, LL29‐30)

According to the participants, VW should be carried out in a controlled manner. This requires choosing the right moment and ending the intervention before the patients become exhausted.

Before actively starting VW, participants discuss the importance of preparatory interventions. These should be carried out to enable the start of weaning and to keep stress on the respirator to a minimum.

I always check to see if something is going in that direction or if I can keep resources that might be available in two or three days when he's no longer bleeding or when his cardiac output is such that I can reduce the medication that is suppressing his cardiac output, which I still need at the moment so that nothing gets damaged, but so that he doesn't become hypoxic when he wakes up and needs more. (GD1, RT01, LL278‐282)

Direct interventions were discussed by participants in such a way that they have a direct, immediate effect on patient's breathing or MV. In addition to spontaneous breathing trials (SBT), this also includes reducing MV support, spontaneous breathing mode on the ventilator, tracheostomy and respiratory stimulation. Treatment of the underlying disease is considered a direct intervention when the lungs are the primary organ affected. In addition, secretion management, targeted sedation with its effect on the respiratory centre, muscle training and early mobilization with their effect on the respiratory muscles are mentioned as direct interventions. Finally, extubation or decannulation is also a direct weaning intervention.

In contrast, indirect interventions support and enable VW but have no direct influence on patients' breathing or MV. These indirect interventions are multifaceted and, in addition to resource conservation, speech and swallowing exercises, also include continuous monitoring, constant evaluation of the weaning approach, interdisciplinary case reviews and the associated prevention of adverse events. Furthermore, the weaning protocol is mentioned as an indirect intervention, which may include instructions not only for SBTs and awakening trials but also for pain management. In addition, sleep management and enabling periods of rest are important interventions, as are treatment of the underlying disease (except respiratory, see direct interventions) and stabilization of haemodynamics. External stimulation of patients, for example, neurological or through music, is also listed alongside ensuring nutrition and maintaining skin integrity. Finally, psychosocial support, for example, through the integration of relatives, is also considered important. In addition to building relationships, this includes ongoing communication with patients, promoting cognition and perception, and ensuring a safe environment.

What a patient needs for weaning […] is a safe environment or at least that the patient feels safe. (GD2, RN10, LL84‐86)

VW is also influenced by external factors that are not part of the program itself. In addition to available time resources, staffing levels and care structures at the facility, these factors also include the premises and equipment of the respective institution.

What materials do you have available (‐) there are so many good things available now also expensive things (‐) whether you want to invest in weaning (‐) in general, ventilator equipment what you have there. (GD3, RN19, LL1134‐1136)

The personality and attitude of the HCPs, communication within the team and management and leadership in the facility also play an important role. Legislation, financing, ethical guidelines and social expectations are also important influencing factors. Finally, the expertise of HCPs depends on their professional experience and the state of knowledge.

I think lack of knowledge, especially among younger employees, plays a big role here, because if […] I'm supposed to start weaning now and he's been given I don't know how many Sufenta boluses during the night because my colleague can't tell the difference between a sedative and an analgesic, then I've got a tough job on my hands. (GD2, RN07, LL154‐158)

After completing VW, the participants emphasized the need for post‐program interventions. They note that a gradual reduction in respiratory support after extubation or decannulation is of key importance.

So not from tube to zero. (GD01, RN06, L64)

HCPs must avoid hard transitions and prevent reintubation with breathing exercises.

It's pointless if he has his tracheostoma his endotracheal tube out, but then ends up being reintubated. (GD2, RN15, LL182‐184)

### 4.4 Links and Relationships

In addition to identifying relevant interventions, outcomes and contextual factors, participants also discussed the links and relationships between these key aspects of VW. The links were divided into eight groups: (1) organization and process, (2) disease and condition, (3) ICU surroundings, (4) breathing and ventilation, (5) sedation, sleep and cognition, (6) nutritional intake, (7) stress development and (8) mobility and muscles.

Several organizational and procedural links were described. The initiation of VW depends on various factors, and the removal of the indication means the termination of the weaning process. Although only frontline workers have a direct influence on weaning, indirect interventions enable direct interventions and influence patients' constitution. In addition, the treatment prior to MV influences the weaning outcome, as do influencing, external factors. Interventions can pursue several objectives, and conversely, the formulated goals determine whether interventions make sense.

Then, of course, that doesn't make sense either, because I naturally use up a lot more resources on the patient than the weaning attempt might actually benefit me. (GD2, RN08, LL260‐262)

Considering disease and condition, participants noted that interventions depend on respiratory parameters and require a safe patient condition. The back and forth reduces the patient's resources; therefore, stable haemodynamics is a precondition for extubation. Pain was identified as a central factor reducing patient's orientation and hindering adequate weaning; pain management, in turn, enables sufficient spontaneous breathing.

If he's in pain, then you don't wean him. (GD1, RN01, L1192)

Several ICU surroundings influence patients' weaning. Although an appropriate working environment is essential and enables VW, noise, for example, hinders successful weaning.

What greatly influences weaning is simply the day‐night rhythm, volume, and calmness. (GD3, RN19, LL1137‐1138)

Participants also identified various relationships with breathing and ventilation. As patients may bloat due to counterpressure and respiratory performance determines the respirator's settings, the patient and respirator must synchronize. In this regard, successful weaning depends on patient's participation. Extubation, in detail, depends on various factors, largely environmental and situational factors. If not successful, prolonged ventilation causes adverse events, and controlled ventilation is linked to muscle loss.

How long has he been ventilated, how severely has his muscle pump or respiratory pump already degenerated as a result of controlled ventilation. (GD2, RN07, LL161‐163)

Furthermore, several relationships involving sedation, sleep and cognition were identified. Participants stated that sedation directly influences patient's breathing pattern and swallowing, but low sedation and critical illness do not contradict each other. Sleep enables rest and relaxation, but an impaired day–night rhythm hinders successful weaning. In addition, patient's alertness, wakefulness and orientation influence the respiratory drive, enable spontaneous breathing and promote swallowing. Although agitation requires sedation, prolonged sedation causes adverse events and delirium hinders sufficient weaning.

The longer and deeper the sedation, the more complications we have, the longer mechanical ventilation takes, and the more delirium we see. (GD2, RN13, LL136‐138)

Nutritional intake was described as supporting both physiological stabilization and psychological well‐being. It is linked to quality of life and patient's muscle loss.

To ensure that he does not lose muscle mass (‐) I need an adequate amount of nutrition. (GD1, RT01, L942)

Furthermore, participants described the development of stress. Pain, delirium, MV and excessive spontaneous breathing cause stress; overall, weaning is therefore associated with stress for patients. At the same time, elevated stress levels were described as inhibiting effective VW.

If the patient is extremely stressed and wakes up completely out of control, then there is no point in even considering weaning. (GD2, RN11, LL174‐175)

Finally, links to mobility and muscles were discussed. Although sedation is not a contraindication for mobilization, too much effort can also be harmful; for example, sitting for too long causes pain. When used carefully, mobilization is a key element: It relieves abdominal pressure, supports the patient's excretion, enables ventilation of different lung areas and prevents weaning failure. According to the participants, muscle strength is related to extubation, and stable seating is a precondition for extubation.

In general, I believe that muscle strength is indeed crucial […] you have to make sure that you don't lose, maintain muscle strength wherever possible (‐) and then rebuild it again (‐) through mobilization, activation. (GD2, RT02, LL576‐579)

## 5 Discussion

### 5.1 Summary of Evidence

Embedded in a larger multi‐methods study, the aim of this sub‐study was to identify key characteristics and mechanisms in VW of adult intensive care patients [9]. To this end, GDs and workshops were held on 3 days with a total of 29 HCPs. Using deductive‐inductive thematic analysis [14], four principal themes were derived: (1) complexity of ventilator weaning, (2) outcomes, (3) interventions and (4) links and relationships.

These four themes, and in particular the first one, support the study's preliminary assumption that VW is a complex intervention according to the MRC Framework [5]. The participants highlighted that different HCPs with varying levels of experience, skills and grades are integral to VW. The heterogeneity of patients, the flexibility and dynamism required, and the numerous interventions, outcomes and contextual factors underscore this complexity. These findings suggest that successful VW cannot be understood as the result of individual interventions. Rather, it emerges from the dynamic interaction between patient characteristics, professional competencies, organizational conditions and continuous adaptation of interventions over time. This systemic perspective reinforces the understanding of VW to be a complex intervention and highlights the importance of considering mechanisms and contextual influences alongside the individual interventions.

The findings of this study are in line with other studies. Various factors and perspectives influence the initiation, performance and course of VW [18]. Data suggest and confirm that HCPs require complex guidelines and thorough training to adequately perform VW [19]. Rather than merely identifying interventions, the findings of this study indicate that their impact depends on the interaction with contextual factors and professional competencies. This extends previous research by emphasizing that VW is to be understood as an adaptive process in which mechanisms operate differently depending on the surrounding context.

In this regard, the training of HCPs is also evident in the nonprogram external factors of this study and shows empirically proven correlations with an improved patient situation: The level of training and educational interventions have an impact on patients' length of stay in hospitals [20], the rate of ventilator‐associated pneumonia [20], the duration of intubation and MV [20, 21], the rate of weaning failure [20] and the reintubation rate [22].

Following Funnell & Rogers [10], nonprogram external factors were assigned to the principal theme of interventions because they represent activities or conditions that influence the delivery and effectiveness of VW without constituting components of the weaning program itself [23, 24]. Examples include staffing level, legislation, financing or patient conditions. Although these factors cannot usually be modified by or during direct patient care, they shape the environment in which program activities are implemented and thereby influence patient outcomes. Thus, nonprogram external factors occupy a dual role within the PT: They represent broader implementation context while simultaneously acting as upstream interventions at the organizational or health system level. Distinguishing these factors from program activities helps to explain why similar weaning activities may result in different outcomes across varying settings. In summary, the interventions incorporate the distinction introduced by Funnell and Rogers between program activities—which are controlled by program implementers—and nonprogram activities—which, as interventions and conditions, cannot be controlled by program implementers but, on an abstract level, define the contextual framework surrounding the program activities.

In addition to these contextual influences, several interventions and outcomes were identified that become relevant at different stages in varying degrees and are important once, intermittently or continuously. The present study structures the key characteristics according to the logic of PT and is not guided by existing classifications such as the ‘Weaning According to a New Definition’ (WIND) study [2], which currently provides the standard terminology (short, difficult and prolonged weaning) for research and clinical practice. This is explained by the fact that program theories are not only more specific than grand theories but also more abstract than practical theories [25, 26]. The differences in the level of abstraction and the objectives make it impossible for program theories to be based exclusively on such classifications.

The connections between the identified interventions, outcomes and contextual factors become clear in the fourth principal theme ‘links and relationships’. These will then be transferred to a separate model in a subsequent publication and presented as a specific PT on VW.

### 5.2 Limitations

Our study was limited to the participation of HCPs in German‐speaking countries who speak and understand German fluently. Participants who are not fluent German speakers were excluded, which may have resulted in a narrowed perspective on the research topic. However, the first step of the study involved three comprehensive reviews of the literature, integrating many international studies into PT.

In addition, no physicians or speech therapists could be recruited to review the theory. Although these professions were represented during the initial theory development, their absence in the revision may have reduced the refinement through an interprofessional perspective. As a result, the revised PT may more strongly reflect nurses' perspectives who constituted the largest group in this study. Therefore, transferability to healthcare settings with different professional roles or interprofessional structures should be interpreted with appropriate caution. Nevertheless, the heterogeneous sample largely reflects routine ICU practice and also included individuals involved in VW in other contexts (e.g., educators).

### 5.3 Recommendations for Further Research

The multitude of interventions, outcomes and contextual factors, as well as their interaction, will be reflected in a revised PT. The correlations formulated therein should be tested as hypotheses in further quantitative studies. In addition, it is advisable to consider fragmented intervention research in terms of embedding multimodal interventions in the context of the ICU and to conduct research accordingly.

### 5.4 Implications for Clinical Practice

The findings have several practical implications for HCPs involved in VW. First, weaning should be considered and re‐evaluated throughout the whole process and not be seen as a single intervention. HCPs should assess weaning indication and readiness on a regular basis, while considering the patients' current condition, their respiratory status and possible contraindications.

Second, HCPs should define and reassess individual and meaningful outcomes for their patients. These may vary and need to be adopted throughout the process. Third, clinical decisions should consider the interaction and timing of several interventions. The findings indicate that gradual and properly dosed intervention bundles are important for patients VW.

Fourth, HCPs should pay attention to modifiable factors that can make VW easier or more difficult. These include appropriate sedation and pain management, prevention of delirium, maintenance of nutrition and muscle mass, proper sleep, mobilization, secretion management and creation of a calm and safe environment. Regular interprofessional communication and reassessment of the VW strategy are also necessary to respond to the dynamics of the situation.

Fifth, the findings indicate that HCPs should not only implement several weaning interventions but also continuously assess the interaction of interventions, patient‐related factors, outcomes and contextual conditions.

## 6 Conclusion

The findings support the understanding of VW in adult intensive care patients as a complex intervention. The four principal themes that emerged in the data analysis are complexity of the weaning process, outcomes, interventions, and links and relationships. The findings indicate that VW is not the result of individual interventions alone but arises as a result of interactions between patient‐related factors, different interventions, outcomes, competencies of professionals and context.

The findings also illustrate that the relevance of the outcomes and interventions varies during the weaning process and that interventions have to be properly timed and adapted to the patient's condition. Factors such as sedation, pain, sleep, cognition, nutrition, mobility, stress, ICU environment, staffing and professional knowledge and skills can help or hinder VW and thus have to be taken into consideration together with ventilator‐related interventions.

Finally, the findings offer empirical evidence for refining the program theory of VW and illustrate the importance of developing individual, integrated and context‐specific care. The identified links between core components need further quantitative testing and investigation within intervention studies to examine their contribution to successful VW.

## Author Contributions

All authors had a substantial contribution to the manuscript and approved it for submission.

## Funding

FS receives a scholarship for his PhD project from the HBG Foundation (https://wissenschaft‐der‐pflege.de/). The HBG Foundation only supports FS financially and has no influence on the approach and content of the dissertation and its publications, including this research article.

## Ethics Statement

Ethics approval for this study has been obtained from the Ethics Committee of the Witten/Herdecke University on 28 April 2025 (Number: S‐109/2025). All study participants had to provide informed consent for this study.

## Consent

The authors have nothing to report.

## Conflicts of Interest

The authors declare no conflicts of interest.

## Supporting information


**File S1:** Exemplary results of the workshops. This supplement contains two illustrations, each showing the results of one workshop. They depict the assignment of the respective interventions and outcomes to the deductive categories of the program theory.

**File S2:** Exemplary codes and quotes for themes. This supplement contains an expanded presentation of data analysis. In addition to the principal and sub‐themes, it also includes individual codes and quotes to make the analysis transparent.

## Data Availability

The data that support the findings of this study are available upon request from the corresponding author. The data are not publicly available due to privacy or ethical restrictions.
