# Supplementary material for: Key Characteristics and Mechanisms in Ventilator Weaning of Adult Intensive Care Patients—A Qualitative Study With Healthcare Professionals
Source: Nurs Crit Care. 2026 Sep 24;31(6):e70678. doi: 10.1111/nicc.70678 (PMC13612930; doi:10.1111/nicc.70678)

Supplementary File S1 – Exemplary results of the workshops

(all workshops and data collection had been conducted in German)

Results of workshop on outcomes (day 2)

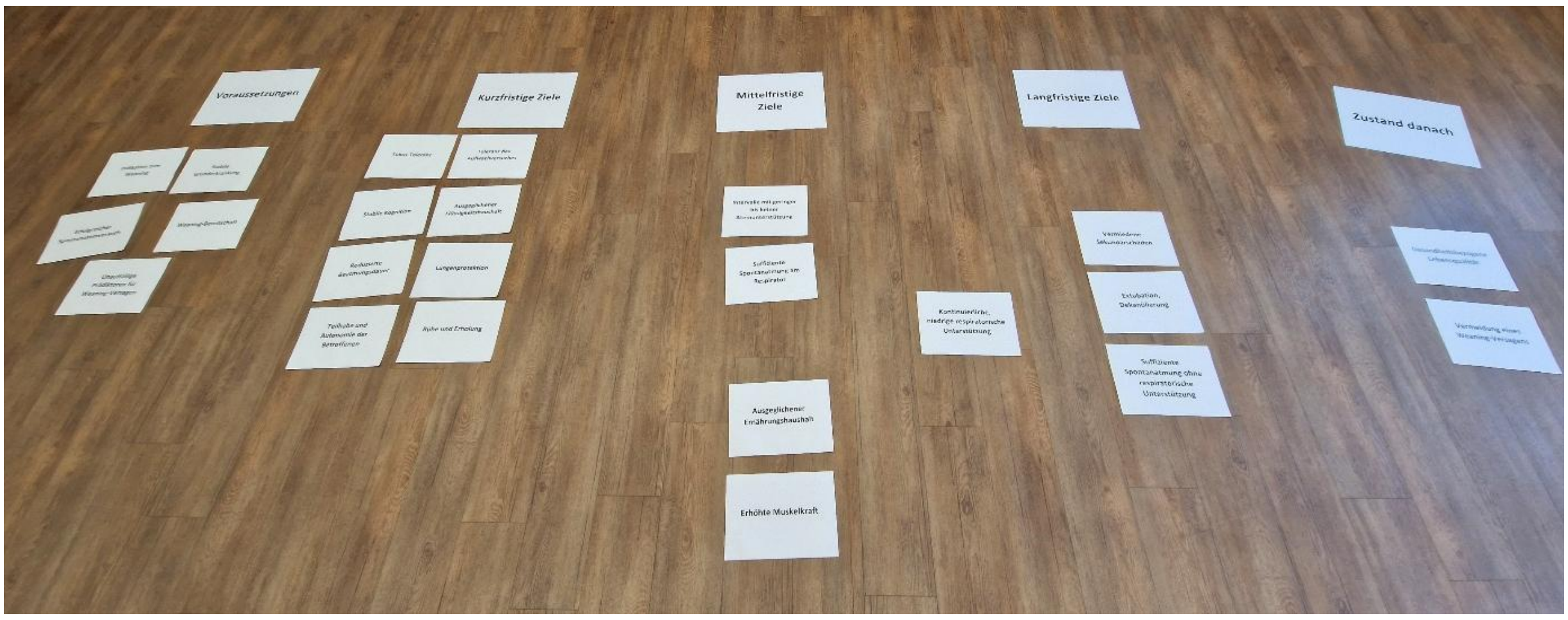

Results of workshop on interventions (day 3)

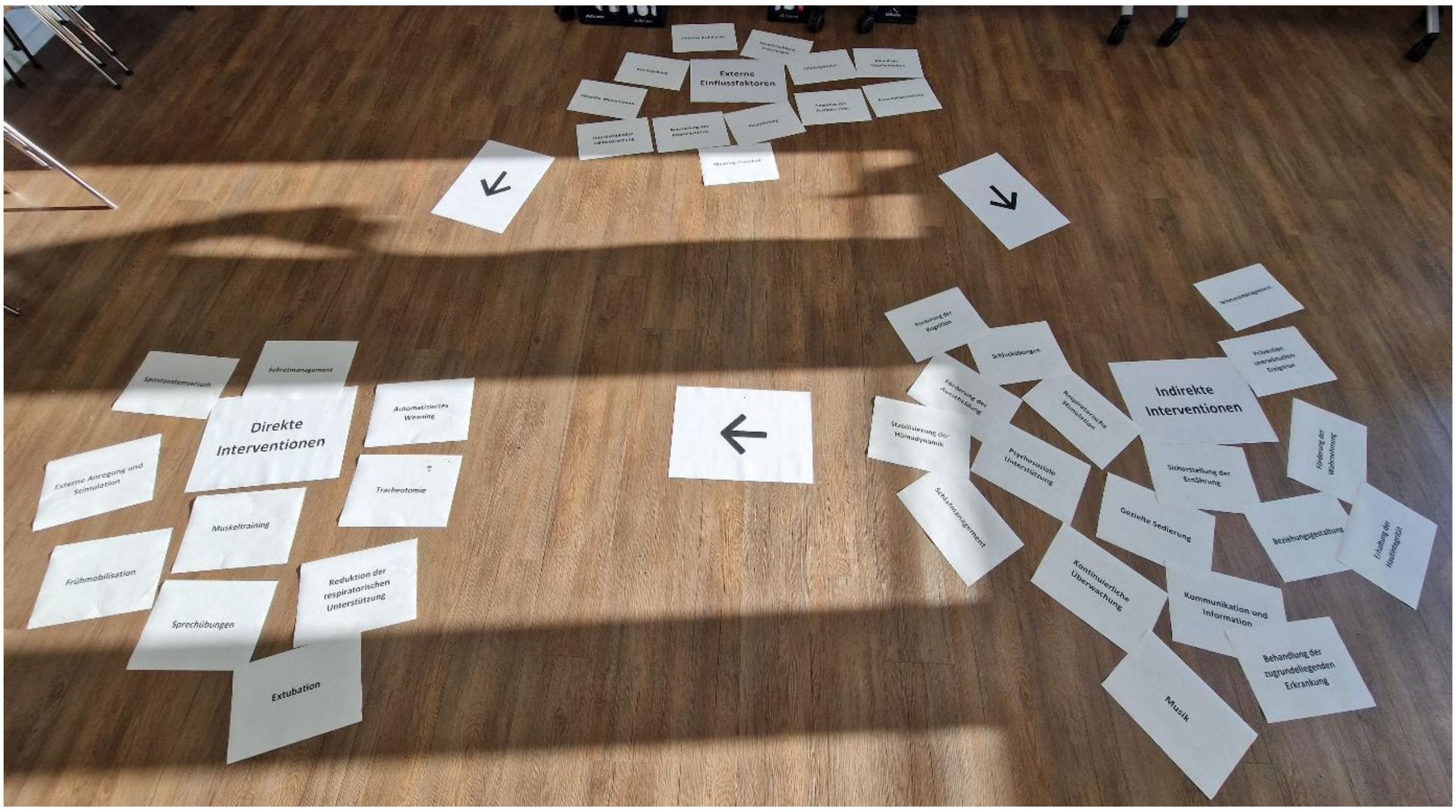

Supplement: Supplementary file 1 [file NICC-31-0-s001.pdf]
