# Supplementary material for: Key Characteristics and Mechanisms in Ventilator Weaning of Adult Intensive Care Patients—A Qualitative Study With Healthcare Professionals
Source: Nurs Crit Care. 2026 Sep 24;31(6):e70678. doi: 10.1111/nicc.70678 (PMC13612930; doi:10.1111/nicc.70678)
Supplement: Supplementary file 2 [file NICC-31-0-s002.pdf]

Supplementary File S2: Exemplary codes and quotes for themes

Legend: FN = Fieldnote; GD = Group discussion; RN = Registered nurse

Principal theme	Theme	Sample codes	Finding or quote
**Complexity of ventilator weaning**	Common understanding within the care team	Linguistic precision is central to weaning	“At 10:07 a.m., RN08 discusses the heterogeneous definition of weaning. The question is raised as to when and where weaning actually begins (e.g., is PEEP reduction already weaning?)” (FN11, LL85-87)
	Delimitation of ventilator weaning	Embedding weaning into the overall care in the ICU	“That's exactly what I wanted to say, that you can't view ventilation in isolation (-) so ventilation is going well, ah no, but he still has to be mobilized in the Thekla and and and or he has to go for a CT scan today” (GD3, RN26, LL469-471)
	Patients’ individual needs and demands	Seeing the person behind the ventilated patient	“That it is an individual (RN02: exactly) that it is an individual who is also permitted to make decisions or who understands why things are not feasible” (GD1, RT01, LL1149-1150)
	Dynamics in the weaning process	Weaning is not always straightforward	“I think small steps at the beginning are, of course, very individual. You have highflyers, yes, but you also have enough who take (-) crazy detours” (GD3, RN25, LL330-332)
	Weighing up in difficult situations	Interventions can also worsen the patient situation	“Because I don't know how he will react to it now, so if I let him become more alert, his pulmonary situation might deteriorate again because he can't quite cope with the ventilation because he stresses himself too much” (GD2, RN09, LL415-418)
	Significance of the factor time	Initiating weaning at an early stage	“As you just said, when weaning begins with intubation, we always tell our participants that weaning actually begins with the decision to intubate, so we try to take a step before that, so to speak.” (GD2, RN08, LL432-435)
	Structure and individualization	Structured approach to weaning is difficult	“Yes, there are few patients who can be treated according to a standard formula, and I think this becomes even more apparent during weaning because there are simply so many different factors at play.” (GD2, RN14, LL561-563)
**Outcomes**	Characteristics of outcomes	Weaning goals change over the course of treatment	“The goals are different from those in prolonged weaning” (GD2, RN10, L359)
	Preconditions	Weaning readiness	“Is he able to walk this path now?” (GD3, LL313-314)
	Immediate outcomes	Successful spontaneous breathing trial	“Check whether he is breathing spontaneously at all” (GD1, RN04, L31)
	Intermediate outcomes	Stable cognition	“In terms of their state of consciousness, patients should naturally be in such a condition that they are able to follow instructions” (GD2, RN08, LL36-37)
	Ultimate outcomes	Extubation	“Yes, I want to get the patient extubated as soon as possible” (GD2, RN08, LL641)
	Postconditions	Health-related quality of life	“That the patient is calm and comes out with little trauma” (GD2, RN11, LL526-527)

**Interventions**	Characteristics of interventions	Correct timing of interventions is essential	"It must be assessed daily whether a spontaneous breathing attempt is advisable, as I believe the timing [to initiate weaning] can easily be overlooked." (GD3, RN19, LL360-362)
	Preparatory interventions	Keeping stress on the respirator low	"That he can participate that he's not completely stressed out now." (GD2, RN15, LL180-181)
	Direct interventions	Secretion management	"Also, prevent secretion problems [...] or perhaps he can use mobilization to move the secretion to another area where he can then clear his throat and swallow it." (GD1, RT01, LL813-817)
	Indirect interventions	Pain management	"Of course, the issue of pain, I mean pain relief [...] that I can even get him to breathe" (GD2, RN07, LL152-159)
	Nonprogram factors	Financing	"This is a major issue for insurances because, ultimately, external financing is needed to cover the costs of these positions [means: respiratory therapists]." (GD1, RN01, LL822-824)
	Post-program interventions	Avoid hard transitions	"So not from tube to zero" (GD1, RN06, L645)
**Links and relationships**	Organization and process	Initiation depends on underlying disease	I think the first thing that needs to happen is for the acute condition that led to intubation and ventilation in the first place to stabilize to such an extent that we can even consider (-) moving towards weaning." (GD2, RT02, LL31-33)
	Disease and condition	Pain inhibits adequate weaning	"If he's in pain, then you don't wean" (GD1, RN01, L1192)
	ICU surroundings	Working environment enables ventilator weaning	"The manager has influence on the setting, etc., but does not directly affect patient care. They merely create the conditions that enable employees to do their work well." (FN06, LL26-28)
	Breathing and ventilation	Prolonged ventilation causes adverse events	"I want to get him off the respirator as soon as possible because we know that the longer he is on it, the higher the risk of him developing pneumonia." (GD2, RN08, LL636-637)
	Sedation, sleep and cognition	Low sedation and critical illness do not contradict each other	"But if you have a safe environment, it makes sense to let your patients wake up even if they are still suffering from an acute illness." (GD2, RN11, LL321-323)
	Nutritional intake	Muscle loss is linked to nutritional status	"To ensure that he does not lose muscle mass (-) I need an adequate supply of nutrition." (GD1, RT01, L942)
	Stress development	Excessive spontaneous breathing causes stress	"If he gets stressed, then we try to find out why [...] because of the respirator or because we let him breathe so heavily." (GD1, RT01, L326-328)
	Mobility and muscles	Stable seating as a prerequisite for extubation	"When I extubate him, I have to bring him into a vertical position, for example, and if he can't manage the vertical position in terms of his circulation (RT01: absolutely), I also have to stop with my weaning." (GD1, RN01, LL284-287)
